# Interference with RUNX1/ETO Leukemogenic Function by Cell-Penetrating Peptides Targeting the NHR2 Oligomerization Domain

**DOI:** 10.1155/2013/297692

**Published:** 2013-06-25

**Authors:** Yvonne Bartel, Manuel Grez, Christian Wichmann

**Affiliations:** ^1^Institute for Biomedical Research, Georg-Speyer-Haus, 60596 Frankfurt, Germany; ^2^Institute for Molecular Medicine, Goethe-University, 60590 Frankfurt, Germany; ^3^Department of Transfusion Medicine, Cell Therapy and Haemostasis, Ludwig-Maximilian University Hospital, 81377 Munich, Germany

## Abstract

The leukemia-associated fusion protein RUNX1/ETO is generated by the chromosomal translocation t(8;21) which appears in about 12% of all *de novo* acute myeloid leukemias (AMLs). Essential for the oncogenic potential of RUNX1/ETO is the oligomerization of the chimeric fusion protein through the nervy homology region 2 (NHR2) within ETO. In previous studies, we have shown that the intracellular expression of peptides containing the NHR2 domain inhibits RUNX1/ETO oligomerization, thereby preventing cell proliferation and inducing differentiation of RUNX1/ETO transformed cells. Here, we show that introduction of a recombinant TAT-NHR2 fusion polypeptide into the RUNX1/ETO growth-dependent myeloid cell line Kasumi-1 results in decreased cell proliferation and increased numbers of apoptotic cells. This effect was highly specific and mediated by binding the TAT-NHR2 peptide to ETO sequences, as TAT-polypeptides containing the oligomerization domain of BCR did not affect cell proliferation or apoptosis in Kasumi-1 cells. Thus, the selective interference with NHR2-mediated oligomerization by peptides represents a challenging but promising strategy for the inhibition of the leukemogenic potential of RUNX1/ETO in t(8;21)-positive leukemia.

## 1. Introduction

Acute myeloid leukemia (AML) is the most common form of myeloid leukemia. In half of all patient-derived AML blasts, chromosomal translocations can be detected leading to the expression of aberrant fusion proteins which are generally not found in normal cells of healthy individuals [1]. Most often, the affected proteins are transcription factors regulating critical steps during hematopoiesis [2]. Their altered function results in the block of cellular differentiation, a general feature of AML.

The chromosomal translocation t(8;21) generates the chimeric protein RUNX1/ETO which is expressed in 12% of all *de novo* AML with 40% of them belonging to the M2 subtype of the FAB (French-American-British) classification [3]. The hematopoietic transcription factor RUNX1 (also known as AML1, CBF*α*2, or PEBP2*α*B) belongs to the family of core-binding transcription factors and is a key regulator of myeloid cell differentiation [4]. As a consequence of the translocation, the DNA-binding domain of RUNX1 (RUNT homology domain, RHD) is fused to almost the entire ETO protein (alternatively named MTG8 or RUNX1T1) which functions mainly as a transcriptional repressor protein [5, 6]. Thus, most RUNX1 target genes are transcriptionally repressed in t(8;21)-positive blasts. Likewise, RUNX1/ETO can act as a positive regulator of gene expression, depending on cofactor recruitment and epigenetic modifications [7]. Expression of RUNX1/ETO in primitive hematopoietic cells leads to increased cell survival, proliferation, and a delay in myelo-erythroid differentiation which certainly contributes to the onset of leukemia development [8]. However, additional genetic alterations are necessary for overt transformation from an initial preleukemic clone [9, 10].

The ETO protein is characterized by four nervy homology regions (NHR1-4) that mediate the interaction with diverse cellular factors such as the nuclear corepressors N-CoR, SMRT, mSIN3A, and histone deacetylases (HDACs) [11, 12]. The NHR2 domain is responsible for the binding to the ETO family members MTGR1 and ETO-2 as well as for homooligomerization [13]. Thereby, RUNX1/ETO generates high molecular weight protein complexes that are critical for the oncogenic potential of the fusion protein [14]. Recent studies suggest the NHR2 domain as one essential ETO domain crucial for RUNX1/ETO-associated leukemogenesis [15, 16]. The crystal structure of a recombinant NHR2 protein revealed an *α*-helical structure with the capacity to build tetramers with a total interaction area of nearly 10,000 Å^2^ [17]. Mutations inside the NHR2 domain that abrogate the formation of oligomers resulted in a complete loss of the oncogenic potential of RUNX1/ETO. We recently demonstrated that a dimeric mutant of RUNX1/ETO, generated by alanine substitution of 5 essential amino acids within the NHR2 domain, is no longer able to transform hematopoietic progenitor cells [18]. Consequently, the selective interference with tetramerization could be a promising strategy to inhibit the oncogenic properties of this fusion protein.

Previously, we have shown that the lentiviral overexpression of a protein containing the entire NHR2 domain efficiently inhibits RUNX1/ETO oligomerization and restores expression of RUNX1 target genes [19]. Proliferation of RUNX1/ETO-dependent cell lines was reduced and the block of differentiation was reverted after expression of the NHR2-only protein, thus allowing for myeloid maturation of blast cells. Furthermore, an increase in apoptosis was measured in treated cells, indicating that the leukemic potential of RUNX1/ETO was efficiently blocked.

Here, we investigated the use of cell penetrating proteins as an alternative approach to deliver the NHR2 domain into RUNX1/ETO-transformed myeloid cells. A TAT-NHR2 polypeptide, TN122, was successfully internalized by human cells and interacted specifically with the ETO protein. In a RUNX1/ETO-dependent myeloid cell line, the consecutive treatment with TN122 inhibited proliferation and increased the rate of apoptotic cells.

## 2. Materials and Methods

### 2.1. Plasmid Construction

Truncated versions of the previously described N89 sequence [19] were generated by PCR and cloned into the SacII sites of the lentiviral vector SiEW. A codon optimized N89 construct was generated by Geneart and also cloned via SacII into the SiEW expression vector.

The TAT fusion constructs were generated by using the bacterial expression vector pSW5 [20], kindly provided by Professor Dr. Winfried Wels (Georg-Speyer-Haus, Frankfurt, Germany). The plasmid contains an N-terminal HIV-1 TAT protein transduction domain and a C-terminal myc- and His(6)-tag. The NLS-containing protein sequences of the NHR2 domain (aa 482-548 in RUNX1/ETO), or the oligomerization domain of BCR (aa 1-72) as a control, were cloned into the vector by PCR using the Kpn I and Hind III cleavage sites. The two cysteines present in the NHR2 sequence were substituted by serine using site-directed mutagenesis (Stratagene kit, Stratagene, La Jolla, CA). A TAT- and NLS-free NHR2 protein was generated using the Nde I/Hind III cleavage sites of the vector. For protein localization studies, a fluorescent protein was generated in which eGFP was inserted into the construct downstream of the NHR2 domain.

### 2.2. Protein Expression and Purification

*E. coli* BL21-CodonPlus (DE3) competent cells were transformed with the expression plasmids. A single clone was used to inoculate an overnight preculture containing ampicillin (100 *μ*g/mL) and glucose (0.8% w/v) in LB medium. The next day, fresh culture medium was inoculated at a ratio of 1 : 10, and the bacteria were grown at 37°C until an OD_600_ of 0.7 was reached. Expression of the proteins was induced with 250 *μ*M IPTG, and the culture was incubated for 4 hours at 30°C. To purify the recombinant proteins, the pelleted bacteria were resuspended in IMAC buffer (20 mM Tris-HCl, 500 mM NaCl, 10% glycerine, 20 mM imidazole, pH 8.0) containing a protease inhibitor cocktail (P8849, Sigma-Aldrich, Taufkirchen, Germany). The cells were lysed by addition of lysozyme (1 mg/mL) and subsequent sonification. For affinity purification of the His-tagged proteins, a HisTrap HP column (GE Healthcare, Uppsala, Sweden) was used. The TAT- and NLS-free NHR2 protein was additionally purified by anion exchange chromatography. The IMAC eluate was diluted 1 : 20 in IEQ running buffer (20 mM Tris-HCl, 3% glycerine, pH 8.0) and purified with a HiTrap HP column (GE Healthcare). The buffer of all protein samples was subsequently exchanged to PBS by using a HiTrap Desalting column (GE Healthcare), and aliquots were stored at −80°C until use.

### 2.3. CD Spectroscopy

CD spectroscopy of the NHR2-containing protein was performed on a Jasco CD spectrometer in a 0.1 cm path-length cuvette using a protein concentration of 50 *μ*M and a temperature of 25°C. The spectrum presented is a cumulative spectrum of three individual scans from 195–250 nm wavelength in increments of 0.1 nm.

### 2.4. Protein Crosslinking

Crosslinking was used to demonstrate the oligomerization status of the recombinant NHR2 proteins as was described before for the BCR oligomerization domain [21]. Briefly, 20 *μ*M of the protein sample diluted in PBS was incubated at room temperature for various time periods with 0.01% glutaraldehyde. The reaction was stopped by addition of SDS-containing 2x Laemmli buffer, and 40 *μ*L of the sample was separated on a SDS-PAGE and stained with coomassie brilliant blue.

### 2.5. Cell Culture and Protein Transduction into Human Cell Lines

293T cells were maintained in DMEM+10% FCS, and Kasumi-1 cells were cultured in RPMI 1640+20% FCS. The TAT fusion proteins were added directly to the cell culture medium of subconfluent 293T cells at a final concentration of 1–5 *μ*M. Optionally, chloroquine and/or heparin were also added. To perform protein transduction into hematopoietic Kasumi-1 cells, the culture medium was exchanged to serum-free X-Vivo 10 medium. In some experiments, the cells were preincubated with dextran sulfate (0.1 or 1 mg/mL) for 3 hours at 37°C and extensively washed with PBS. Cells were then seeded at a density of 4 × 10^5^ cells in 500 *μ*L into a 24-well plate, and the cell-penetrating proteins were added to the medium at a final concentration of 2–8 *μ*M. In some experiments, a TAT-HA2 peptide (5 *μ*M) was cotransfected with TN122 to improve peptide uptake. All cells were treated with trypsin for 10 minutes at 37°C and extensively washed before further analysis.

### 2.6. Peptide Synthesis

The TAT-HA2 peptide was ordered from BioLux (Stuttgart, Germany) in retroinverso sequence as described before [22]. To increase peptide stability, D-enantiomers were incorporated for the last two amino acids on both termini.

### 2.7. Coimmunoprecipitation and Western Blot Analysis

Binding of the different NHR2 constructs to ETO protein was analyzed by cotransfection of 293T cells with vectors coding for the respective proteins. After two days, cells were lysed in NP40-containing buffer. NHR2-containing proteins were immunoprecipitated using an antibody directed against the Flag-tag of the proteins (anti-Flag M2, Santa Cruz, CA). The immune complexes were bound to protein A/G-agarose (Santa Cruz) and washed extensively. The coprecipitated ETO protein was then analyzed by western blotting using a polyclonal antibody for ETO (C-20, Santa Cruz). EGFP was detected to check for the transfection levels. Secondary HRP-coupled antibodies were obtained from Santa Cruz, and western blots were developed using the Pico ECL reagent (Thermo Scientific, MA).

The TAT-NHR2 fusion protein TN122 was added twice (0 and 24 h) in a final concentration of 5 *μ*M and in the presence of 20 *μ*M chloroquine to a 293T cell line stably expressing the NHR2-containing protein NC128 [19]. 14 hours later, the cells were treated with trypsin, washed, and lysed in NP40-containing buffer. Immunoprecipitation of the intracellularly expressed NC128 protein was performed using an antibody directed against the Flag-tag of the protein. The immune complexes were analyzed by western blotting using an antibody directed against the myc-tag of TN122 (anti-myc 9E10, Santa Cruz) or against eGFP (Roche, Mannheim, Germany) and actin (I-19, Santa Cruz) as a control.

### 2.8. Confocal Laser Scanning Microscopy

For localization studies, an eGFP-containing TN122 protein was used and added to 293T cells in a final concentration of 2 **µ**M in the presence of 20 **µ**M chloroquine. 24 hours later, a trypsin digestion was performed, the cells were extensively washed, and the cellular membrane was stained by a rhodamine-conjugated wheat germ agglutinin (W-849, Molecular Probes, Invitrogen, Darmstadt, Germany) for 30 minutes at 37°C. The sample was covered with ProLong Gold (SigmaAldrich), and the living cells were analyzed with a confocal laser scanning microscope (TCS-SL, Leica, Wetzlar, Germany) using the 63x oil immersion objective.

### 2.9. Flow Cytometry

The percentage of transduced Kasumi-1 cells was determined by the expression of the marker gene eGFP. For flow cytometry, cells were washed, resuspended in PBS, and analyzed on a FACS-Calibur (Becton Dickinson, Heidelberg, Germany). To investigate levels of CD34 expression, transduced Kasumi-1 cells were stained with an APC-conjugated CD34-antibody (BD Biosciences) for 20 minutes at room temperature in the dark prior to flow cytometry analysis. 10,000 cells were measured and analyzed using the CellQuest Pro software.

The percentage of eGFP-stained cells after addition of cell penetrating NHR2-eGFP proteins was also analyzed by flow cytometry. 7-amino-actinomycin D (7AAD) and allophycocyanin (APC)-conjugated Annexin V (both from BD Biosciences) were used to determine the percentage of dead cells.

## 3. Results

### 3.1. Definition of the Minimal NHR2 Sequence Required for RUNX1/ETO Inhibitory Function

**Figure 1:**
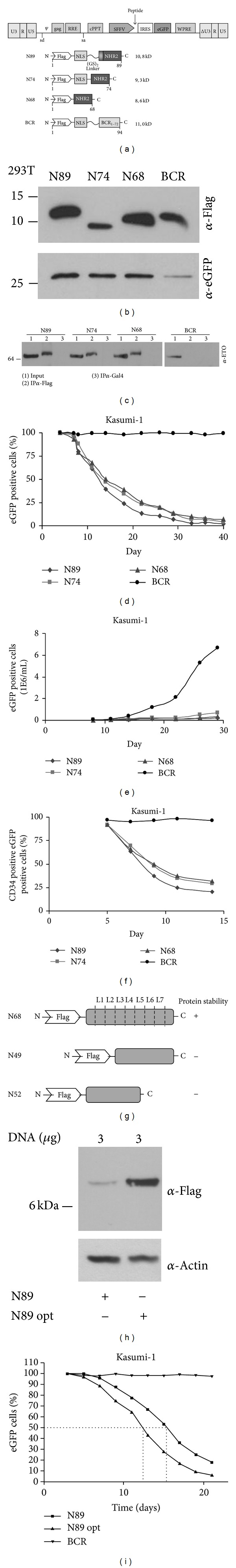
Definition of the minimal NHR2 sequence required for RUNX1/ETO inhibitory function. (a) Lentiviral vector SiEW and tested NHR2-containing constructs. (b) Overexpression of the individual proteins in 293T cells and detection of the Flag-tagged constructs by Western blotting. The marker protein eGFP serves as a loading control. (c) Binding of all NHR2-containing proteins to ETO. Co-transfection of 293T cells with the respective constructs together with a plasmid coding for the ETO protein. Immunoprecipitation of the individual protein complexes was performed with an anti-Flag antibody. (d–f) Cellular effects following lentiviral expression of the different NHR2 constructs in Kasumi-1 cells. Time course of the percentage of eGFP-positive cells (d), growth curve of the transduced cells (e), and time course of the expression of the progenitor cell marker CD34 for transduced Kasumi-1 cells (f). (g) Scheme of N68 based NHR2 deletion forms. Indication of the 7 alpha-helical loops L1-L7 of the NHR2 domain. (h) Comparison of N89 and codon-optimized N89 expression levels by western blotting. (i) Percentage of transduced cells in cocultures expressing N89 and the codon-optimized version thereof.

In order to define the minimal length of the NHR2 proteins required for optimal inhibition of RUNX1/ETO, we generated mutants of the previously described inhibitory protein N89 [19] by deleting the C-terminal flanking region of the NHR2 domain (N74) and the NLS sequence (N68) (Figure 1(a)). A control protein contained the oligomerization domain of the breakpoint cluster region (BCR) protein (aa 1-72) instead of the NHR2 domain. These constructs were then cloned into the lentiviral vector SiEW. Proper expression of the proteins was verified in lysates from transfected 293T cells by western blotting (Figure 1(b)). Next, binding of the polypeptides to ETO protein was investigated by immunoprecipitation of the Flag-tagged proteins from co-transfected 293T cells. All three NHR2-containing proteins were able to interact with the ETO protein (Figure 1(c)), while the BCR control failed. For analyzing the inhibitory potential of the deletion mutants on RUNX1/ETO function, Kasumi-1 cells were transduced with viral vectors and the growth of transduced (i.e., eGFP expressing) cells investigated by flow cytometry analysis. The expression of each of the three NHR2-containing proteins led to a decrease in the relative amount of transduced cells over time as indicated by expression of the marker protein eGFP (Figure 1(d)). This was due to an inhibition of cellular growth of transduced Kasumi-1 (Figure 1(e)), while the nontransduced cells in the culture were not affected in their growth kinetics. Contrary to this, cells transduced with the BCR control construct did not show any signs of growth inhibition. Furthermore, in contrast to the BCR control peptide, expression of the NHR2-containing constructs in Kasumi-1 cells induced a decrease in the expression of the progenitor cell marker CD34 over time (Figure 1(f)). This indicates that expression of the NHR2 sequence was, at least in part, able to overcome the differentiation block mediated by RUNX1/ETO. Although, in general, all three NHR2-containing constructs displayed inhibitory potential, N89-expressing Kasumi-1 cells showed a slightly faster decrease in the amount of transduced cells (Figure 1(d)) as well as a faster and even stronger decrease in the percentage of CD34 positive cells (Figure 1(f)) arguing for a stronger inhibitory potential of N89 in comparison to the deletion mutants N74 and N68. Further shortening of the NHR2 core amino acids, the seven alpha-helical loops, led to instability of the polypeptides N49 and N52 (Figure 1(g)). To demonstrate a dose-dependent effect of inhibitor peptide expression, we compared N89 with a codon-optimized version with increased intracellular peptide levels (Figure 1(h)). Indeed higher N89 expression correlated to a faster net decrease of transduced cells arguing for a dose dependency of NHR2 inhibitor peptides (Figure 1(i)).

### 3.2. Design of Constructs, Expression, Purification, and Analysis of Recombinant Proteins

**Figure 2:**
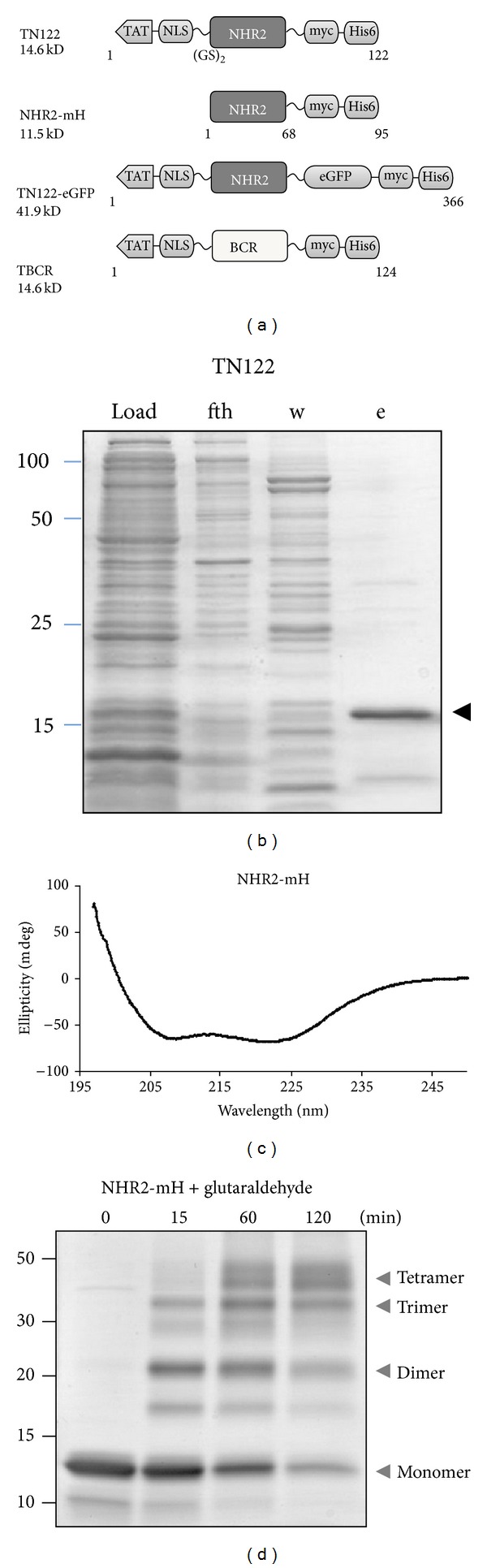
Purification and analysis of recombinant NHR2 containing polypeptides. (a) Schematic representation of the constructs used in this study. *TAT*, protein transduction domain of HIV-1 TAT protein; *NLS*, nuclear localization signal of SV40. (b) Purification of the recombinant NHR2 containing polypeptide TN122 by nickel affinity chromatography. SDS-PAGE and coomassie brilliant blue staining of the bacterial lysate (load), the flow through (fth), the washing fraction (w), and the eluate (e). The arrow indicates the purified TN122 protein. (c) CD spectroscopy of the NHR2 protein (NHR2-mH) at 25°C in PBS reveals an *α*-helical structure. (d) Glutaraldehyde crosslinking of the NHR2 protein for the indicated incubation times at room temperature and subsequent SDS-PAGE analysis. The arrows indicate the different oligomerization states of the crosslinked NHR2 proteins.

Cell-penetrating polypeptides were generated by linking the protein transduction domain of the HIV-1 TAT protein (YGRKKRRQRRR) to the N-terminus of the polypeptides used in this study. The results obtained with the NHR2-deletion mutants (Figure 1) and our previous results [19] indicate that the complete NHR2 domain is required for an effective inhibitory function of NHR2 peptides. Although a construct lacking the NLS also does inhibit cell growth of RUNX1/ETO-dependent Kasumi-1 cells (Figure 1), the NLS domain was included in all constructs for maximal effects. Therefore, the NHR2 polypeptides were based on the N89 sequence containing a nuclear localization signal (NLS) from SV40, a flexible GSGS linker, and the complete NHR2 domain (aa 482–548 in RUNX1/ETO). In addition, a myc- and a histidine-tag were included at the C-terminus for detection and purification of the recombinant polypeptides, respectively, resulting in the TAT-NHR2 polypeptide TN122 (Figure 2(a)). For cellular localization studies, a TN122-eGFP fusion construct was also generated. As a control polypeptide, the oligomerization domain of BCR was used to substitute for the NHR2 domain in the TN122 construct. The proteins were expressed in *E. coli* and purified from the bacterial lysates under native conditions by immobilized metal ion affinity chromatography (IMAC). After optimization of the protocol, a relatively pure protein fraction of TN122 was obtained (Figure 2(b)).

Before testing the recombinant polypeptides in cell culture, we analyzed the structural properties of the NHR2 domain within the NHR2 containing polypeptides. This was necessary since deviation from the expected *α*-helical structure could affect the biological properties of the fusion polypeptides. For these studies, we used a protein that lacked the TAT and NLS sequences and consisted by two thirds of the NHR2 domain (NHR2-mH). This recombinant protein was selected because of its high purity after ion exchange chromatography, as determined by analytical ultracentrifugation (purity of >90%, data not shown). The secondary structure of NHR2-mH was investigated by CD spectroscopy revealing the typical curve shape of an *α*-helical protein (Figure 2(c)). From ellipticity measurements at 222 nm, the helical content of the protein was calculated [23] and revealed a value of 64%. Assuming that the myc- and the histidine-tag are unstructured, this value indicates that the NHR2-mH polypeptide was properly folded. It has been previously described that the isolated NHR2 domain forms tetramers [17]. To investigate the oligomerization state of the recombinant NHR2-mH polypeptide, crosslinking experiments were performed using glutaraldehyde as a crosslinker to preserve the rigidity of the protein structure after denaturation. Analysis of the crosslinked sample on SDS-PAGE revealed the existence of monomers, dimers, and tetramers (Figure 2(d)). With prolonged incubation times, the proportion of tetramers increased at the expense of the lower oligomer forms. This observation verifies that the recombinant NHR2 polypeptides retained their ability to form tetramers *in vitro*.

### 3.3. Cell-Penetrating NHR2 Proteins Transduce Mammalian Cells and Bind to ETO Protein Sequences

**Figure 3:**
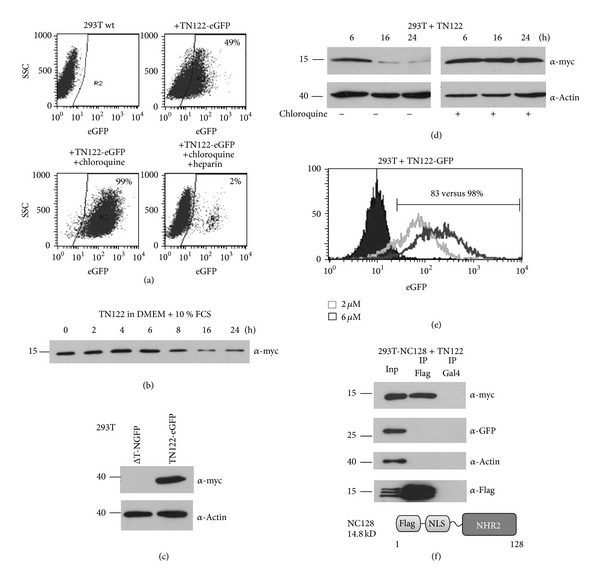
Protein transduction into 293T cells and interaction of recombinant TN122 with the NHR2 domain. (a) Protein transduction of 1 **µ**M TN122-eGFP into 293T cells in the presence or absence of 100 *μ*M chloroquine and addition of 10 *μ*M heparin. After 24 h, the cells were treated with trypsin (10 min, 37°C) and analyzed for the percentage of eGFP positive cells by flow cytometry. (b) Incubation of 1 **μ**M TN122 for various times in serum-containing medium at 37°C and analysis of the stability of the protein by western blotting. (c) TAT-mediated protein transduction. Incubation of 293T cells with either 10 *μ*M ΔT-NGFP or 2 *μ*M TN122-eGFP in the presence of 100 *μ*M chloroquine for 4 h. Thereafter trypsin treatment and western blot analysis of the myc-tagged proteins. (d) Influence of chloroquine on protein transduction. The 293T cells were incubated with 1 *μ*M TN122 in the presence or absence of 20 *μ*M chloroquine for the indicated time, treated with trypsin, and analyzed for the myc-tagged TN122 protein by western blotting. (e) Concentration dependency of protein transduction. Incubation of 293T cells with 2 *μ*M (light gray) or 6 *μ*M (dark gray) TN122-eGFP for 3 h and trypsin treatment followed by flow cytometry. The content of eGFP positive cells is indicated. (f) Binding of the recombinant cell-penetrating NHR2 polypeptide to ETO protein sequences. Protein transduction of 2 × 5 **μ**M TN122 (at 0 and 24 h) in the presence of 20 *μ*M chloroquine into 293T cells that stably express the NHR2-containing polypeptide NC128. The cells were treated with trypsin 14 hours after the last addition of the protein. The Flag-tagged NC128 was immunoprecipitated and the co-precipitated TN122 detected by western blotting. The blot was also stained for eGFP and actin to verify the specificity of the interaction.

The transduction potential of TN122 was investigated by adding the recombinant TN122-eGFP protein to the culture medium of 293T cells followed by flow cytometry for the assessment of eGFP positive cells. To guarantee that only successfully internalized proteins were detected, a digestion with trypsin was performed before FACS analysis to cleave all TAT-proteins bound to the outer side of the plasma membrane. After an incubation time of 24 hours, almost 50% of the cells showed the presence of the eGFP marker protein (Figure 3(a)). The addition of chloroquine, an inhibitor of endosome acidification and lysosomal protein degradation, to the cell culture medium at a concentration of 100 *μ*M increased the uptake and intracellular stability of the TN122 polypeptide resulting in a 100% of the cells expressing eGFP at 24 hours after addition of the polypeptide. Therefore, it can be assumed that, initially, all of the adherent cells were successfully transduced with TN122-eGFP but in the absence of chloroquine, the protein was slowly degraded by lysosomes leading to a decrease in the content of eGFP positive cells over time. To further control for a TAT-mediated uptake of TN122-eGFP, heparin was added to the cell culture medium resulting in a complete block in uptake as described previously [24]. The TAT domain attaches to the cells mainly by binding to heparan sulfate proteoglycan (HSPG) on the outer surface of the cells [25]. An excess of soluble heparin competes with HSPG for binding to the TAT domain and thus inhibits the TAT-mediated cellular internalization of proteins. Kinetic studies on the stability of TN122 showed that the polypeptide was stable for at least 8 hours in the cell culture medium at 37°C and could be detected even at 24 hours after the initial addition (Figure 3(b)). Western blotting experiments revealed that successful protein transduction was only achieved with the TN122 construct, while an NHR2 protein lacking the TAT-PTD was not able to transduce the cells even when high concentrations were used (Figure 3(c)). Internalized TN122 was detected for at least 24 hours in cell lysates prepared from transduced 293T cells in the presence of chloroquine, while no TN122 was detectable 16 hours after protein transduction in the absence of chloroquine (Figure 3(d)). For the intracellular stabilization of the transduced protein, a minimal concentration of 20 *μ*M chloroquine was necessary as determined by titration experiments (data not shown). By using increasing concentrations of TN122-eGFP, the efficiency of protein transduction measured by flow cytometry could be enhanced (Figure 3(e)).

We also investigated whether TN122, upon protein transduction, was able to interact with ETO inside the cells. For this purpose, we used a 293T cell line stably expressing the Flag-tagged NHR2 polypeptide NC128 [19]. NC128 expressing cells were incubated twice with 5 *μ*M TN122 in the presence of chloroquine. Coimmunoprecipitation experiments revealed that TN122 was indeed able to interact with NC128 (Figure 3(f)). This binding was specific since neither the highly expressed eGFP protein nor actin was co-precipitated with NC128. Also, no unspecific binding to the antibody was detected when cellular extracts from wild type 293T were used in this assay (data not shown).

### 3.4. Requirements for TAT-Mediated Protein Transduction into Myeloid Kasumi-1 Cells

**Figure 4:**
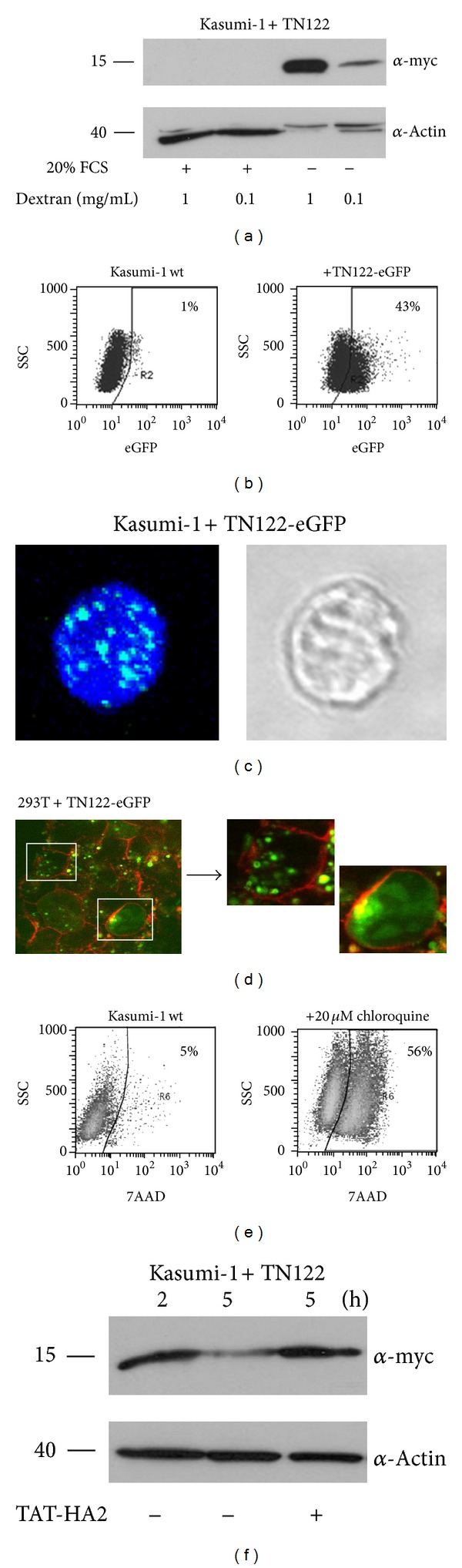
Cell-penetrating NHR2-containing polypeptides transduce myeloid Kasumi-1 cells. (a) Effect of serum and dextran on protein transduction efficiency into hematopoietic cells. Preincubation of Kasumi-1 cells for 3 hours in dextran (0.1 or 1 mg/mL) containing medium, extensive washing of the cells and incubation with 2 *μ*M TN122 for 4 hours in the presence or absence of 20% FCS. Successfully transduced protein was detected by Western blotting after trypsin treatment of the cells. (b) Incubation of Kasumi-1 cells with 6 *μ*M TN122-eGFP in serum-free X-Vivo 10 medium for 3 h, trypsin treatment and flow cytometry analysis to determine the efficiency of protein transduction. Percentages correspond to the percentage of eGFP positive cells. (c) Intracellular localization of TN122-eGFP in Kasumi-1 cells. Incubation of the cells with 4 *μ*M TN122-eGFP in X-Vivo 10 medium for 1 h, trypsin treatment, fixation and permeabilization, DNA staining with Toto3, and subsequent CLSM analysis (63x magnification). (d) TN122-eGFP is captured inside endosomes upon protein transduction. Incubation of 293T cells with 2 *μ*M TN122-eGFP in the presence of 20 *μ*M chloroquine for 24 h, trypsin treatment, staining of the unfixed cells with a rhodamine-coupled wheat germ agglutinin (red), and CLSM analysis. (e) Cytotoxicity of 20 *μ*M chloroquine on Kasumi-1 cells. The percentage of dead cells was measured by flow cytometry using 7-amino-actinomycin D (7AAD) after a 24 hours incubation of the cells in chloroquine-containing medium. (f) Endosomolytic TAT-HA2 increases the intracellular stability of transduced proteins. Cotreatment of Kasumi-1 cells with 3 *μ*M TN122 in the presence or absence of 5 *μ*M TAT-HA2 for different times, trypsin treatment and detection of the myc-tagged TN122 in the cellular lysates.

Based on the successful transduction of TN122 into mammalian 293T cells, we next investigated the cell culture conditions required for an efficient transduction of the TAT fusion proteins into hematopoietic cell lines. In contrast to the results obtained with the adherent cell line, uptake of TN122 by the RUNX1/ETO-expressing Kasumi-1 cell line could only be observed in the absence of serum (Figure 4(a)). The preincubation of the cells with dextran sulfate could further enhance the efficiency of TN122 internalization in a dose-dependent manner as determined by increased amounts of TN122 detectable in cellular lysates prepared from transduced Kasumi-1 cells (Figure 4(a)). Dextran sulfate is a structural analogue of HSPG and is able to act as an artificial attachment receptor for TAT proteins in cell lines that express low levels of HSPG [26]. However, serum-free RPMI medium as well as prolonged incubation times with dextran sulfate had a negative effect on the viability of Kasumi-1 cells (data not shown) and were therefore not used for further experiments. As an alternative, X-Vivo 10 medium was used, and the concentration of TN122 was increased to compensate for the poor uptake of the TAT proteins by the myeloid cell line. Flow cytometry analysis of cells treated with the TN122-eGFP protein revealed that the efficiency of protein transduction into Kasumi-1 cells, as determined by the percentage of eGFP positive cells under optimal conditions, was lower than that observed for 293T cells (compare Figures 3(a) and 4(b)). At a concentration of 6 **µ**M TN122-eGFP and an incubation time of 3 hours, eGFP fluorescence was detected in 43% of the cells.

In order to investigate the localization of the internalized TAT fusion proteins, Kasumi-1 cells were incubated with TN122-eGFP and analyzed by confocal laser scanning microscopy (CLSM) 24 hours after transduction. The fluorescent protein was detected exclusively in vesicular structures (Figure 4(c)). Assuming that the TN122-eGFP proteins enter the cells by endocytosis [27], these vesicles were most likely endosomes. To verify this observation, protein localization studies were also performed using 293T cells. 24 hours after protein transduction in the presence of chloroquine, TN122-eGFP was found to localize mainly to large vesicles, confirming the observations made in Kasumi-1 cells. Only a minor fraction of TN122-eGFP was localized to the cytosol under these conditions (Figure 4(d)). The use of similar cell culture conditions for Kasumi-1 cells revealed that a concentration of 20 *μ*M chloroquine was toxic for Kasumi-1 cells and therefore not suitable for long-term experiments (Figure 4(e)). With the intention to increase the intracellular stability of the transduced proteins in Kasumi-1 cells, we tested a fusion peptide derived from the influenza virus protein hemagglutinin-2 (HA2). The HA2 peptide has been shown to destabilize the lipid membrane of endosomes upon acidification, thereby enabling the release of molecules from endosomes into the cytoplasm [28]. Therefore, the HA2 sequence was fused to the TAT domain of HIV-1 to generate a cell-penetrating endosomolytic peptide that could be used to release internalized TAT proteins from the endosomes [22]. Consequently, the co-treatment of Kasumi-1 cells with TN122 and TAT-HA2 resulted in an intracellular stabilization of the internalized TN122 as reflected by the high amount of polypeptide detected in western blots 5 hours after treatment of the cells. In contrast, the amount of TN122 in cellular lysates of Kasumi-1 cells was significantly decreased 5 hours after the addition of the protein in the absence of TAT-HA2, presumably because the majority of TN122 was retained within the endosomes and thus subjected to protein degradation via the lysosomal route (Figure 4(f)). This result indicates that the HA2 peptide was effective in releasing TN122 from the endosomes and could be used as an alternative to chloroquine to perform functional studies of TN122 in Kasumi-1 cells.

### 3.5. TN122 Negatively Affects Proliferation of Kasumi-1 Cells and Decreases Cellular Viability

**Figure 5:**
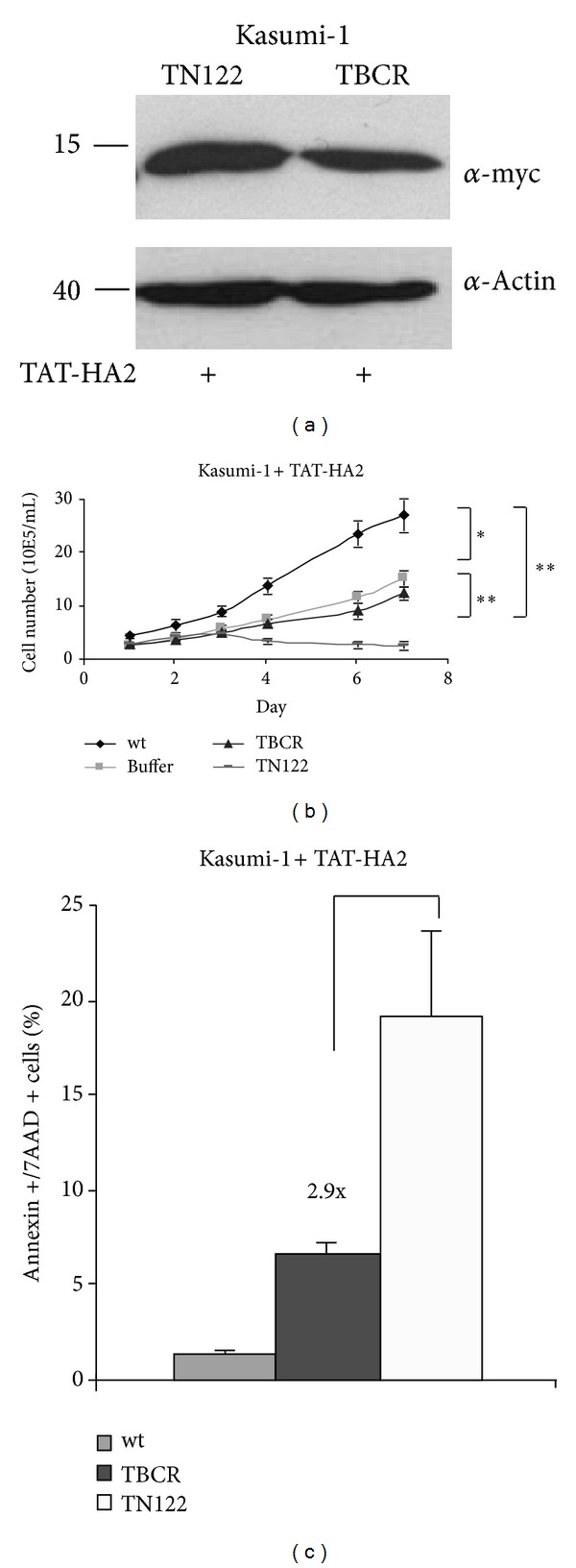
Cellular effects of TN122 on the proliferation and viability of Kasumi-1 cells. Incubation of Kasumi-1 cells for 7 days in X-Vivo 10 medium with daily cotreatment of the cells with 8 *μ*M TN122 or TBCR as a control protein and 5 *μ*M TAT-HA2. (a) Western blot detection of both cell-penetrating proteins in the cellular lysates 5 hours after the last addition of the proteins. (b) Analysis of the proliferation rates of treated Kasumi-1 cells. At day 0, 2 × 10^5^ c/mL were seeded, and cell numbers were measured daily by trypan blue staining. The values are mean values with the corresponding standard deviation of the experiment carried out in triplicates. Data were statistically analyzed using two-tailed student's *t* test for unpaired samples; *P* < 0.05 was considered significant (∗) and *P* < 0.01 highly significant (∗∗). (c) Analysis of the percentage of apoptotic cells by flow cytometry at day 7. Shown is the percentage of cells that are double positive for Annexin V and 7AAD. The values are mean values with the corresponding standard deviation of the experiment carried out in duplicates.

The effects of the cell-penetrating NHR2 protein TN122 on Kasumi-1 cells were investigated after daily administration of 8 *μ*M TN122 plus 5 *μ*M TAT-HA2 to the cells over a period of 7 days (Figure 5). To demonstrate the specificity of the cellular effects mediated by TN122 on the RUNX1/ETO growth-dependent cell line, we used in parallel a cell-penetrating peptide containing the oligomerization domain of BCR (TBCR) as a control protein. The successful internalization of TN122 and TBCR by Kasumi-1 cells was demonstrated by western blotting (Figure 5(a)). Under the influence of TN122, Kasumi-1 cells showed a strongly decreased proliferation rate which was evident three days after start of the protein treatment (Figure 5(b)). Although a negative effect of the buffer itself on cell proliferation became evident during these experiments, the strong reduction in proliferation rate observed with TN122 cannot be explained by the influence of the buffer alone as the reduction in cell proliferation in the presence of TN122 was highly significant. In contrast to TN122, the BCR containing polypeptide did not affect proliferation of Kasumi-1 cells beyond the effect caused by the buffer alone (Figure 5(b)). The negative effect of the cell-penetrating NHR2 protein on the cellular viability was further analyzed via flow cytometry by measuring the percentage of apoptotic cells. At day seven after start of TN122 treatment, a significant increase in the percentage of Annexin V and 7AAD double positive Kasumi-1 cells was detected. This effect was clearly caused by TN122, as the apoptotic effect mediated by the control polypeptide TBCR was 3-fold lower than that observed with TN122, indicating that induction of apoptosis by the cell-penetrating NHR2 polypeptide on RUNX1/ETO-dependent Kasumi-1 cells was specific (Figure 5(c)).

## 4. Discussion

The current treatment of acute myeloid leukemia with t(8;21) translocation is based mainly on the use of cytotoxic drugs, especially anthracyclines and cytarabine, with a median survival time from first diagnosis of 2-3 years and a 5-year overall survival of less than 40% [29, 30]. Due to the lack of specificity and selectivity, this treatment is in most cases associated with severe side effects that can be fatal particularly for older patients. An alternative strategy that specifically targets the leukemic cells is therefore highly desirable. Consequently, numerous studies have concentrated on the development of molecular therapies targeted at tumor-relevant functions of leukemia-specific oncoproteins [31, 32]. Whereas the clinical relevance of inhibitors of histone deacetylases and demethylating agents to revert the block of myeloid differentiation seems to be limited [33], better results were achieved using tyrosine kinase inhibitors such as gleevec to decelerate the enhanced proliferation of the blast cells. Originally developed for the treatment of BCR/ABL positive chronic myeloid leukemia, gleevec is also effective for several constitutively active mutations of c-kit found in numerous t(8;21) positive patients [34]. However, under the influence of kinase inhibitors, the development of escape mutations in the kinase domain leading to drug resistance has been reported repeatedly [35]. Obviously, novel specific therapies are still required.

Leukemias with t(8;21) are addicted to the permanent expression of the RUNX1/ETO fusion protein [19, 36]. In order to eliminate the transformed cells, inhibition of crucial protein-protein interactions could therefore be a suitable strategy for a targeted therapy against RUNX1/ETO. We have previously shown that the leukemogenic potential of RUNX1/ETO can be inhibited by interference with tetramerization of the chimeric protein using proteins containing the NHR2 oligomerization domain, which were expressed intracellularly in leukemic cells [19]. However, for a therapeutic approach, the application of viral vectors *in vivo* is difficult due to the lack of efficient targeting. As an alternative delivery strategy, we therefore investigated whether the protein transduction technology could be utilized to directly deliver the inhibitory polypeptides to the leukemic cells. Several studies have demonstrated the feasibility of this approach also for hematopoietic cells. For example, a truncated mutant of nucleophosmin coupled to the TAT domain was found to inhibit proliferation and induce apoptosis in preleukemic stem cells [37]. Another group could show that cell-penetrating peptides derived from AF4 were able to specifically induce necrosis in cells expressing the ALL-associated fusion protein MLL/AF4 [38]. More recently, it was demonstrated that a constitutively active mutant of the transcription factor FOXO3 fused to the TAT domain induced apoptosis in leukemic cell lines and reduced viability of primary chronic lymphoid leukemia (CLL) cells [39]. In another promising approach, the coiled-coil domain of BCR was used to generate the cell-penetrating peptide TAT-CC in order to interrupt BCR/ABL oligomerization [40]. The authors could show that the peptide interacted with BCR/ABL endogenously expressed in leukemic cell lines and, as a consequence, led to a decrease in cell growth and induction of apoptotic death specifically in cells expressing the leukemia-associated fusion protein. For RUNX1/ETO, Racanicchi et al. used protein fragments derived from the RUNX1/ETO corepressor N-CoR to disrupt this essential protein-corepressor interaction. As a result, expression of RUNX1/ETO repressed genes was restored leading to myeloid differentiation of leukemic cell lines [41]. As N-CoR has multiple functions in development, homeostasis and prevention of disease, N-CoR-derived peptides may interfere with essential cellular functions of the protein, and thus, their therapeutic application must be taken with caution. Furthermore, recent studies have shown that an alternatively spliced isoform of RUNX1/ETO lacking the C-terminal N-CoR binding domain coexists with full length RUNX1/ETO in patients and strongly induces leukemia development in mice [42]. Consequently, peptides targeted to the N-CoR-RUNX1/ETO interaction domain may not be fully effective in t(8;21) leukemias expressing the truncated form of RUNX1/ETO. We therefore propose that targeting the oligomerization domain of RUNX1/ETO, which is crucial for the activity of both, the full-length as well as the truncated protein, could be a more valuable approach.

Here, we show that by fusion to the TAT protein transduction domain, recombinant NHR2 polypeptides could successfully be internalized by mammalian cells. As reported elsewhere for TAT mediated protein transfer [22], uptake of TAT-NHR2 polypeptides occurred most likely by macropinocytosis, a specialized form of endocytosis, because they were found to localize to endosome-like vesicles throughout the cytoplasm. We do not expect the eGFP-tag used for intracellular localization studies to influence cellular traffic since in our previous studies, an NHR2-eGFP fusion construct had the same antiproliferative effect on Kasumi-1 cells compared to an NHR2-only protein [19]. Moreover, successful protein transduction and biologic activity has been demonstrated with much larger proteins like *β*-galactosidase [43]. However, the size of the proteins does influence the time required for protein transduction [44]. The trapping of TAT fusion proteins in endosomes is a common observation made by several groups [45], and the escape of these proteins to the cytosol seems to be the rate limiting step in the protein transduction process that has to be optimized to fully exploit this methodology [46]. Chloroquine treatment effectively inhibited degradation of the proteins, thereby increasing their intracellular stability. The occurrence of large vesicular structures in which the internalized proteins are trapped is also attributed to the usage of chloroquine that induces lysosomal dilatation. It is believed that a high concentration of the TAT domain in the vesicles is able to destabilize the endosomal membrane, thus allowing a certain amount of the protein to escape from the endosomes and carry out its therapeutical function in the cell [47]. In agreement with this, the successfully internalized TN122 interacted with ETO protein sequences inside the cells.

In comparison to the adherent cell line, the amount of internalized TN122 that was detected in the hematopoietic Kasumi-1 cells was significantly lower. The poorer protein transduction efficiency of the suspension cell line is most likely related to different expression levels of HSPG at the cell surface that serves as a binding molecule for the TAT domain. Whereas HSPG is expressed at high levels on most adherent cells, only low amounts are found on hematopoietic cells [48]. Although it has been reported that myeloid cells from healthy donors do not express HSPG [49], a low but clear expression of HSPG was detected in myeloid leukemia cell lines as well as in leukemic blasts of AML patients [50]. However, for efficient *in vivo *targeting, a tumor-associated antigen fused to TAT-NHR2 may be required, as was successfully demonstrated for a TAT-p53-derived protein targeting cells overexpressing the CXCR4 receptor [51].

Other studies have reported the use of chloroquine to successfully inhibit lysosomal degradation of internalized cell membrane-penetrating proteins [20, 52]. However, in these cases, cells were generally treated for short time periods, most likely to avoid cytotoxic reactions associated with chloroquine that is known to inhibit cellular growth and viability by blocking lysosomal hydolases, arresting autophagy, activating the p53 pathway, and inducing apoptosis. Thus, in order to perform repetitive protein transduction of TN122 into Kasumi-1 cells over an extended time period, we used the TAT-HA2 peptide as an alternative to chloroquine. Coadministration of TAT-HA2 and TN122 to the cells resulted in a prolonged detection of internalized TN122, most likely mediated by increased endosomal escape of TAT-NHR2 proteins that consequently were no longer subjected to lysosomal degradation. The internalized TN122 polypeptides were found to bind to ETO as well as to inhibit proliferation and increase apoptosis in RUNX1/ETO positive cells, resembling the effects observed after lentiviral-mediated expression of NHR2 peptides in Kasumi-1 cells [19]. In order to control the specificity of these effects, we made use of the BCR tetramerization domain which has a very similar structure to that of the NHR2 domain with its four *α*-helical monomers building the tetramer. With the lentiviral vector system, the utilization of BCR as a proper control could be demonstrated (Figure 1 and [19]). Contrary to this, a monomeric mutant of the NHR2 domain (m7) introduced by the Bushweller group [17] disrupts protein stability and could therefore not be used as a control because its expression and purification as a cell-penetrating protein failed. The influence of TN122 on Kasumi-1 cells was specific since a similar polypeptide containing the BCR oligomerization domain did not affect cell growth and vitality beyond the effects observed with the buffer alone. The buffer used for purification of the cell-penetrating proteins includes imidazole that has toxic effects on cells. It cannot be ruled out that small amounts are still present in the buffer after dialysis against PBS causing the growth inhibitory and apoptotic effects observed with both, the buffer alone and the BCR control. However, the potency of the TN122 effects was reduced compared to that observed after lentiviral transduction and expression of NHR2-containing polypeptides. These observations are not surprising as the confocal microscopic analysis of protein-transduced cells revealed that the majority of the internalized TN122 protein was localized in vesicles, and only a small proportion was able to escape to the cytosol. Obviously, the amount of NHR2 polypeptides released from the endosomes after internalization was not sufficient to completely abolish the leukemogenic potential of RUNX1/ETO. Since the efficiency of protein transduction dependents on the concentrations is used, we tested different concentrations of TN122 for growth inhibition in Kasumi-1 cells. While the effects with 1 *μ*M TN122 were modest, 5 to 10 *μ*M were found to be optimal leading to a significant reduction in cell growth. Higher protein concentrations did have an unspecific toxicity due to impurities present in the solvent as mentioned before and therefore were not further used.

The efficiency of protein transduction into hematopoietic cells strongly depends on the cell line used. Racanicchi et al. reported that a TAT fusion protein designed to interfere with the binding of PML/RAR*α* to the corepressor molecule N-CoR in PML/RAR*α*-expressing NB4 cells was able to induce full myeloid differentiation in the presence of vitamin D3. In contrast, a similar fusion protein designed to block the binding of RUNX1/ETO to N-CoR merely led to a partial induction of differentiation in the RUNX1/ETO positive cell line SKNO-1 [41]. Besides considerations on the uptake efficiency of cell-penetrating peptides by different cell types, the cellular localization of the TAT fusion proteins may also have a profound influence on the biological effects of the transduced proteins. RUNX1/ETO tetramers are found in high molecular weight protein complexes of >2 MDa that are stabilized by several protein-protein interactions. Thus, it is doubtful that these complexes can be destroyed efficiently by the NHR2 polypetides in the nucleus. More likely, the NHR2 polypeptides interfere during the synthesis of new RUNX1/ETO molecules by competing with the full-length protein for the assembly into RUNX1/ETO tetramers. Probably, high concentrations of the inhibitory polypeptides will be required inside leukemic cells to efficiently interfere with RUNX1/ETO complex assembly and oncogenic activity.

Because the size of the construct has a critical influence on efficiency of protein transduction [53], we tested several truncated versions of the previously described NC128 protein [19] and chose N89 as the shortest protein with the full inhibitory potential. Further deletions of the construct that could increase transduction efficiency were not as efficient in its inhibitory function as N89. When using the TAT-PTD an additional NLS sequence might be dispensable [54], and the tags included for purification and western blot detection are not required to achieve biological efficiency of the protein. However, our data indicate that the entire NHR2 domain is necessary for the full inhibitory potential since the deletion of only seven amino acids inhibits the binding of the construct to RUNX1/ETO [19]. Recently, we were able to show that five amino acids in the NHR2 sequence are critical for tetramerization of the leukemic protein and are involved in its oncogenic potential [18]. Because these hot spots for tetramer formation are located at the outer edges of the NHR2 sequence, it is mandatory to include the whole 59 amino acids of the NHR2 domain.

Altogether, the functional effect of TN122 is limited. However, this strategy serves as a proof of principle and indeed is the first report showing that a peptide-based protein domain interference strategy can be regarded as a potential way to interfere with RUNX1/ETO tetramerization and oncogene function. Current limitations of the protein transduction approach are mainly in terms of efficiency and specificity. In order to increase the intracellular stability of the proteins, usage of D-amino acids is possible that would, however, require chemical synthesis of the peptides. Transduction efficiency of the proteins is mainly hampered by their improper release from endosomes. To overcome this hurdle, several different endosomolytic peptides of viral or bacterial origin have been described with the capacity to destabilize endosomal membranes [55]. Specificity of the therapeutic protein for the cancer cells might be achieved by the incorporation of tumor-associated targeting peptides. Of course, also a combination therapy of the cell-penetrating peptides with inducers of differentiation like vitamin D3 and/or valproic acid is possible. As an alternative to protein transduction, NHR2-containing proteins might be administered by liposomes or other nanoparticles [56]. However, further approaches will concentrate on the development of peptide mimetica and small molecular weight inhibitors with increased cellular uptake and efficiency that are able to interfere with NHR2 tetramer formation. As we recently described, indeed disruption of tetramers into dimmers is sufficient to block RUNX1/ETO oncogenic function [18].

Our results indicate that the NHR2 domain of RUNX1/ETO is a challenging but promising target for a molecular intervention in t(8;21)-positive leukemia. Since oligomerization of chimeric proteins seems to be essential for their leukemogenic activity [57], we further propose that interference with oligomerization could be a general principle towards a targeted therapy in leukemia. Indeed, the value of this approach has already been demonstrated for the BCR/ABL fusion protein using a BCR-derived TAT-coiled-coil peptide [40]. Likewise, it should also provide an attractive treatment option for RUNX1/ETO-mediated leukemia.
